# Interaction of Supramolecular Congo Red and Congo Red-Doxorubicin Complexes with Proteins for Drug Carrier Design

**DOI:** 10.3390/pharmaceutics13122027

**Published:** 2021-11-28

**Authors:** Anna Jagusiak, Katarzyna Chłopaś, Grzegorz Zemanek, Izabela Kościk, Irena Roterman

**Affiliations:** 1 Jagiellonian University Medical College, Faculty of Medicine, Chair of Medical Biochemistry, Kopernika 7, 31-034 Krakow, Poland; kchlopas@su.krakow.pl (K.C.); grzegorz.zemanek@uj.edu.pl (G.Z.); izabela.koscik@uj.edu.pl (I.K.); 2 Jagiellonian University Medical College, Faculty of Medicine, Department of Bioinformatics and Tele-Medicine, Lazarza 16, 31-530 Krakow, Poland; myroterm@cyf-kr.edu.pl

**Keywords:** supramolecular self-assembled ribbon-like structures (SRLS), Congo red (CR), doxorubicin (Dox), bovine serum albumin (BSA), immunoglobulin light chain λ (Lλ), heat aggregated immunoglobulins (HAI), dynamic light scattering (DLS), elution volume (V_e_)

## Abstract

Targeted immunotherapy has expanded to simultaneous delivery of drugs, including chemotherapeutics. The aim of the presented research is to design a new drug carrier system. Systems based on the use of proteins as natural components of the body offer the chance to boost safety and efficacy of targeted drug delivery and excess drug removal. Congo red (CR) type supramolecular, self-assembled ribbon-like structures (SRLS) were previously shown to interact with some proteins, including albumin and antibodies complexed with antigen. CR can intercalate some chemotherapeutics including doxorubicin (Dox). The goal of this work was to describe the CR-Dox complexes, to analyze their interaction with some proteins, and to explain the mechanism of this interaction. In the present experiments, a model system composed of heated immunoglobulin light chain Lλ capable of CR binding was used. Heat aggregated immunoglobulins (HAI) and albumin were chosen as another model system. The results of experiments employing methods such as gel filtration chromatography and dynamic light scattering confirmed the formation of the CR-Dox complex of large size and properties different from the free CR structures. Electrophoresis and chromatography experiments have shown the binding of free CR to heated Lλ while CR-Dox mixed structures were not capable of forming such complexes. HAI was able to bind both free CR and CR-Dox complexes. Albumin also bound both CR and its complex with Dox. Additionally, we observed that albumin-bound CR-Dox complexes were transferred from albumin to HAI upon addition of HAI. DLS analyses showed that interaction of CR with Dox distinctly increased the hydrodynamic diameter of CR-Dox compared with a free CR supramolecular structure. To our knowledge, individual small proteins such as Lλ may bind upon heating a few molecules of Congo red tape penetrating protein body due to the relatively low cohesion of the dye micelle. If, however, the compactness is high (in the case of, e.g., CR-Dox) large ribbon-like, micellar structures appear. They do not divide easily into smaller portions and cannot attach to proteins where there is no room for binding large ligands. Such binding is, however, possible by albumin which is biologically adapted to form complexes with different large ligands and by tightly packed immune complexes and heat aggregated immunoglobulin-specific protein complex structures of even higher affinity for Congo red than albumin. The CR clouds formed around them also bind the CR-Dox complexes. The presented research is essential in the search for optimum solutions for SRLS application in immuno-targeting therapeutic strategies, especially with the use of chemotherapeutics.

## 1. Introduction

Drugs designed to reach molecular targets, among which monoclonal antibodies and kinase inhibitors are most frequently used, are the basis of modern therapy. Targeted immunotherapy is also expanded to simultaneous delivery of drugs, including chemotherapeutics. Immuno-targeting, defined as the use of immunological specificity directed to target connected with therapy, is still the subject of many investigations. Design and development of efficient carriers of anti-inflammatory and anticancer drugs are now extensively studied in order to increase the effectiveness and safety of the targeted therapies [1,2,3,4,5].

Self-assembled structures presented in this work are the group of compounds (polyaromatic molecules of an elongated shape with appropriately located polar groups) showing a tendency to self-associate via non-covalent interactions thus creating greater supramolecular systems. This phenomenon is also observed during the formation of microtubules or biological membrane structures. Some of these systems form elongated structures referred to as self-assembled ribbon-like structures (SRLS). These kinds of structure have the potential to be a part of systems delivering chemotherapeutics to cancerous tissue by immuno-targeting. 

This is possible because of their ability to selectively interact with immune complexes. SRLS are examples of a novel type of protein ligand, as they bind to proteins via different interactions than the classic type [6]. SRLS systems bind to proteins at sites of local structural instability caused by unfolding conditions or function-derived structural changes in the protein molecule. The binding of SRLS to antigen-antibody complexes, with simultaneous lack of binding of free antibodies, can serve as an example. The described interaction is a foundation for using those compounds in immuno-targeting [7]. At the same time, SRLS systems can intercalate other molecules, including drugs, forming co-micellar systems [8].

Previous research has shown that SRLS can be applied in vivo as potential drug carriers. Such systems were easily bound to immune complexes formed in the body and then were gradually eliminated. Immune complexes are highly complex systems and their structural analysis is difficult. Conformational changes observed in Lλ under sub-denaturing conditions, which mimic those in antigen-complexed antibodies, contribute to CR binding. This is the reason why immunoglobulin light chain (Lλ) heated to 45 °C was used for the research on interaction between SRLS and antigen-bound antibodies as a model system [9]. Heat aggregated immunoglobulins such as immunoglobulin G (HAI) were investigated as another immune complex model system. One more protein that can be used in targeted therapies, albumin, was also found to bind supramolecular ligands. Some therapeutic agents, especially of cationic nature (like the widely used chemotherapeutic doxorubicin) cannot bind to albumin directly. Thus, albumin can be used in targeted therapy other than in combination with negatively charged carriers. In particular, when SRLS bind drugs, co-micellar structures are formed that can interact with albumin which allows for effective drug delivery and also protects the body against uncontrolled drug action [6,10,11,12,13,14,15,16].

The standard example of SRLS is Congo red (CR), which forms assemblages of elongated shape, with a high level of plasticity [6]. CR is a molecule with polyaromatic rings of elongated planar symmetric structures, substituted by amino and sulfonic groups [17,18]. The presence of sulfonic groups makes the structure polyanionic at neutral pH. Symmetrical charge arrangement and hydrophobic interaction between groups in the central part of the molecules, which is noncovalent, make the ribbon-like structure stable but also guarantees its high plasticity [6]. In the presented work, only CR was used as a model because its properties as SRLS are well known. In the future, it can be replaced by other, more biocompatible compounds with similar properties (e.g., Evans blue) [19,20,21].

The special property of supramolecular CR is that it is able to interact with proteins especially those containing β structure fragments. The mechanism of interaction of supramolecular ribbon-like CR with protein is different from classic protein–ligand interaction in the protein active site. Such complexes can be formed on the condition that protein β-sheet part is at least partially destabilized, which allows for function-derived conformational rearrangement. Thus, susceptibility of CR structures to deformations allowing the best fitting to the protein binding site is the important condition for optimal CR-protein binding. Since there is no defined, specific amino acid sequence that binds CR and the supramolecular ligand is capable of changes due to its plasticity, a variety of proteins are able to form such complexes. Proteins binding to supramolecular CR are native proteins (normal and pathological) as well as proteins treated with denaturing factors [6,22,23].

Examples of native CR binding proteins include molecules in which supramolecular CR binding site arises from function-derived intramolecular strain. Antibodies belong to such group of proteins. In antibodies, antigen binding induces some structural changes [24,25]. Native, antigen-unbound immunoglobulins G do not form complexes with CR but gain this ability upon antigen binding. In the presence of CR, the effect of antibody–antigen interaction enhancement was observed [26]. The property of selective CR binding shown by antigen-bound antibodies gives opportunities for its application in targeted drug delivery [6]. The model system for such interaction was developed as an isolated immunoglobulin light chain (Lλ) heated to 45 °C or immunoglobulin G heated to 63 °C (HAI). Upon heating, the protein is destabilized. The N-terminus is locally unfolded, which opens up the V domain. The same phenomenon takes place when antibodies are bound to the antigen. At the Fab ends associated with the antigen, the beta structure rich polypeptide is revealed and those structures are penetrated by and bound to CR. CR-Dox co-micelles can bind at the same region. However, it should be underlined that it is just a model system, and the real mechanism of CR-Dox co-micelle binding to immunological complexes might be different than that described as binding to the cavity emptied by the N-terminal chain fragment [9]. To our knowledge, there is no other research on the binding of CR complexed with drugs to heated Lλ or HAI.

Another example of CR-binding native protein is serum albumin. This universal carrier of many hydrophobic compounds (especially anionic) can bind CR as single molecules as well as a supramolecular ligand thanks to its structure with the binding cavity [6,11,12,13,14,15,16]. Albumin is a protein adapted to the transport of a variety of anionic compounds that can bind CR without preliminary structure change. The molecule of serum albumin binds up to 16 CR molecules and at most nine molecules of Evans blue, which has a similar structure to CR but shows weaker self-association properties [10]. Until now there have been no investigations of albumin ability to bind CR-drug complexes. There have also been no studies on drug transfer from a carrier such as albumin to the immune complex (presented here as the HAI model).

**Figure 1:**
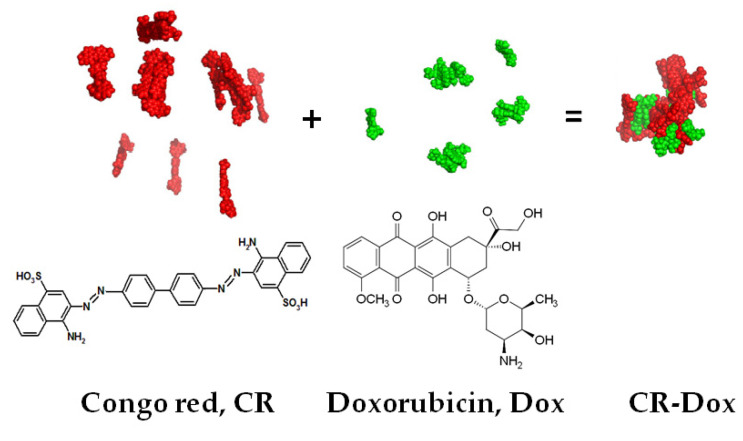
Structure of Congo red and Doxorubicin and the supramolecular complex CR-Dox.

SRLS shows the ability to form complexes with planar molecules including chemotherapeutics, such as doxorubicin [8]. Doxorubicin (Dox) is one of the most effective drugs used in the therapy for many types of cancer [27]. At the same time, it is a highly cardiotoxic drug, and its use can result in the inhibition of hematopoiesis and gastrointestinal disorders [28,29]. Despite this, it is still widely used because of its high effectiveness and wide spectrum of anticancer effects. That is why further investigations of doxorubicin carriers are very important as they can improve the efficiency of its delivery and reduce its toxic effects. Earlier experiments have shown that doxorubicin binds to supramolecular ribbon-like CR structure (Figure 1) [30,31,32,33].

Previously published research on the structure of free CR and CR-Dox complex showed large differences between these systems. Positively charged Dox binds to negatively charged CR and the complex migrates faster than free CR towards the anode during electrophoresis [34]. The absorption spectra of CR and its complex with Dox also differ (hypochromic effect). CR-Dox complexes have increased size compared to free CR, which was confirmed using DLS and molecular modeling methods [8]. The fuzzy oil drop model, applied in the previous study, aimed to detect binding sites for supramolecular ligands in albumin (particularly between its pseudo-symmetrical fragments) as well as in V domains, indicated that complexation of dye molecules led to the formation of a stable supramolecular structure, anchored between antibodies that participate in the immune complex [35].

The main goal of the present analysis was to compare the capability of drug binding mediated by CR to proteins: albumin, heated light chain, which is a model system of antibody bound to antigen, and antibodies bound to surface-immobilized antigens. Using DLS and molecular sieve methods, sizes of the free supramolecular system (CR) and its complex with a drug (CR-Dox, molar ratio 2:1) were compared at various concentrations of components and ionic strength of buffers. Subsequently, the interaction of free CR and its drug-bound complexes (CR-Dox) with proteins was investigated. The properties of SRLS, particularly their ability to bind drugs, antibodies in immune complexes and albumin justify research into the application of the described systems in targeted therapy (immuno-targeting). This research with the use of carriers specifically interacting with some proteins is important for targeted transport of drugs to the desired parts of the body, where inflammation or neoplastic process continues.

## 2. Materials and Methods

### 2.1. Materials

Congo red (CR, 96% purity, Aldrich Chemical Company, Inc., Milwaukee WI 53233, USA), doxorubicin hydrochloride (Dox, 98% purity, Sigma-Aldrich, Co., 3050 Spruce Street, St. Louis, MO 63103, USA), bovine serum albumin (BSA, 96% purity, Sigma-Aldrich, Co., 3050 Spruce Street, St. Louis, MO 63103, USA), and immunoglobulin light chain λ dimer (Lλ) were obtained from the urine of a patient with multiple myeloma. After salting out and dialysis it was purified on Sephacryl S300 column (Pharmacia). Immunoglobulin G was obtained from Baxter Healthcare Corporation, Hyland Division Glendale, CA 91203, USA. All other reagents used were of analytical grade and were purchased from commercial sources.

### 2.2. Methods of CR-Dox Preparation

CR-Dox complexes of the 2:1 molar ratio were used because in the previously optimized CR:Dox ratio doxorubicin is completely bound to CR. CR-Dox complexes were created by adding 2 volumes of preheated (2 min. at 100 °C) Congo red (1.43 mM CR in 0.05 M Tris/HCl buffer pH 7.4, 0.154 M NaCl) to 1 volume of 1.43 mM Dox dissolved in the same buffer. The mixture was incubated for 15 min at room temperature. The cohesion of the CR molecules forming the ribbon-like structure is not high, but in the presence of alkaline doxorubicin complexes are formed and its cohesion increases significantly. The complexes were passed through a Sephadex G-200 column to remove unbound components.

For DLS analysis of the effect of concentration on the hydrodynamic diameters of the analyzed probes, different concentrations were used. The final concentrations of higher concentration probes were: CR (1.43 mM), Dox (0.715 mM), and CR:Dox (molar ratio = 2:1, CR = 1.43 mM, Dox = 0.715 mM). All probes were dissolved in 0.05 M Tris/HCl. pH 7.4 buffer with 0.154 M NaCl. 

The final concentration of lower concentration probes were: CR (0.31 mM), Dox (0.15 mM) and CR:Dox (molar ratio = 2:1, CR = 0.31 mM, Dox = 0.15 mM). All probes were also dissolved in 0.05 M Tris/HCl. pH 7.4 buffer with 0.154 M NaCl. 

For DLS analysis of the effect of buffer ionic strength on the hydrodynamic radius, different concentrations of NaCl in the buffer were used (0.05 M Tris/HCl, pH 7.4 buffer with 0.154 M NaCl or 0.3 M NaCl). The final concentrations of probes in this experiment were lower: CR (0.31 mM), Dox (0.15 mM) and CR:Dox (molar ratio = 2:1, CR = 0.31 mM, Dox = 0.15 mM).

### 2.3. Methods of CR and CR-Dox Binding with Protein

#### Complexes of CR and CR-Dox with Proteins

CR and CR-Dox complexes with three different types of acceptor proteins were analyzed: 1. partly unfolded immunoglobulin chain Lλ; 2. partly unfolded immunoglobulin G (HAI); 3. plasma albumin.

1. The immunoglobulin light chain λ (Lλ)

Partly unfolded Lλ (dimer) was used as a model protein that binds CR or CR-Dox complexes according to the same mechanism as the one observed in antigen-complexed antibodies, where natural structural destabilization is caused by intramolecular constraints evoked by simultaneous interaction with two antigenic determinants [25]. Lλ was isolated from the urine of a patient with multiple myeloma. Complexes were formed by mixing partially unfolded Lλ (obtained by 20 min. incubation at 45 °C (sub-denaturing conditions) with a ten-fold molar excess of CR or CR-Dox complexes (2:1 molar ratio). Under such conditions the N-terminal polypeptide loop of the Lλ undergoes local structural destabilization creating the binding site for 4 CR molecules (per monomer) [9].

2. Heat aggregated immunoglobulin G (HAI)

Human immunoglobulins G at a concentration of 10 mg/mL (0.05 M PBS buffer) were heated for 20 min at 63 °C. The aggregate dissolves upon the addition of 100-fold molar excess of CR. To remove free or weakly bound dye molecules the protein-dye complex was filtered through Sephadex G-200.

3. Albumin

Albumin is a model system for studying the CR and CR-Dox interaction with a typical carrier protein. Bovine serum albumin (BSA) in 0.05 M Tris/HCl buffer, 0.145 M NaCl, pH 7.4 was mixed and incubated (15 min.) with CR or CR-Dox at 10-fold molar excess of CR to BSA, and 2:1 ratio of CR to Dox.

### 2.4. Characterization of Free CR-Dox Complexes or CR-Dox Bound to Albumin, Lλ Light Chain, or HAI

#### 2.4.1. Dynamic Light Scattering (DLS)

Hydrodynamic radii of CR, Dox, and CR-Dox complexes were measured by using the dynamic light scattering (DLS) method (detector Zetasizer Nano ZSP, Malvern, United Kingdom) with laser incident beam at λ = 633 nm and a fixed scattering angle of 173°. For the measurements, dispersants with the following parameters of viscosity and refractive index were used: (1) Tris/HCl 0.05 M with NaCl 0.3 M; viscosity 0.9208 cP; Refractive Index = 1.334; and (2) Tris HCl 0.05 M with NaCl 0.154 M; viscosity 0.9068 cP; Refractive Index = 1.332. Each measuring probe was incubated inside the instrument (3 min/25 °C). A measurement comprised 5–9 repetitions each of which was an average of 15 records measured for 9 s. Outliers were rejected from analysis and the results were averaged.

#### 2.4.2. Gel-Filtration Chromatography (BioGel P-10 and BioGel P-300)

Elution volume (V_e_) analysis for fractionation within a different size range was performed on 100 mm Bio^®^Spin columns (BioRad, Hercules, CA, USA). Columns were filled with 4 mL of BioGel P-10 in 0.01 M PBS buffer, pH 7.4 for supramolecular compound solutions (CR, CR-Dox). For complexes of CR and CR-Dox with proteins (BSA and Lλ), the BioGel P-300 in 0.01 M PBS buffer, pH 7.4 was used. The flow rate reflects the size of the complex (or the supramolecular entity). 80 µL of the sample was loaded onto the column. Then, it was rinsed with 0.01 M PBS buffer, pH 7.4.

The presence of the protein in the eluate was detected by dot-staining with bromophenol blue, CR concentration was determined spectrophotometrically and Dox fluorometrically.

#### 2.4.3. Gel Electrophoresis

Electrophoresis was carried on 1% agarose plates (in 0.06 M sodium barbital buffer, pH 8.6) at 160 V for about 40 min. The position of CR-complexes was recorded, plates were then fixed with picric acid and the excess of CR was removed by reduction with sodium dithionate followed by staining for protein with bromophenol blue. 

#### 2.4.4. TEM

Transmission Electron Microscopy (TEM; JEOL JSM-7500F) was used to evaluate the structure of the HAI-CR complexes. Samples for TEM were prepared by heating the aqueous solution of immunoglobulin G at 63 °C for 20 min. After gradual cooling, the CR solution was added (the CR was labeled with AgNO_3_). The resulting complexes of HAI-CR were purified from the excess of unbound CR using a thin layer chromatography on Sephadex G200. The procedure allows for obtaining well-dispersed HAI loaded with supramolecular CR “clouds”. For TEM analysis, 1 μL of suspension was applied to the surface of the copper grid (300 mesh) and dried in vacuum.

### 2.5. Methods of Analysis of Competition between BSA and HAI for Binding of the CR-RhoB Complexes (Congo Red-Rhodamine B) or CR-Dox (Congo Red-Doxorubicin) Complexes

Additionally, BSA, BSA-CR complex (10:1), and BSA-CR-Dox (or BSA-CR-RhoB) complexes (CR-Dox or CR-RhoB ratios were 1:1, 2:1, or 5:1) were prepared. To remove excess CR unbound with the triple complex, gel-filtration chromatography on BioGel P-300 medium was used for all systems. HAI was added to a part of the sample to observe the transmission of CR and CR-Dox complex from the initial complexes with BSA.

## 3. Results

### 3.1. Characterization of CR-Dox Co-Micelles

#### 3.1.1. DLS Analysis of CR-Dox Complexes—The Effect of Concentration on the Complex Size

**Figure 2:**
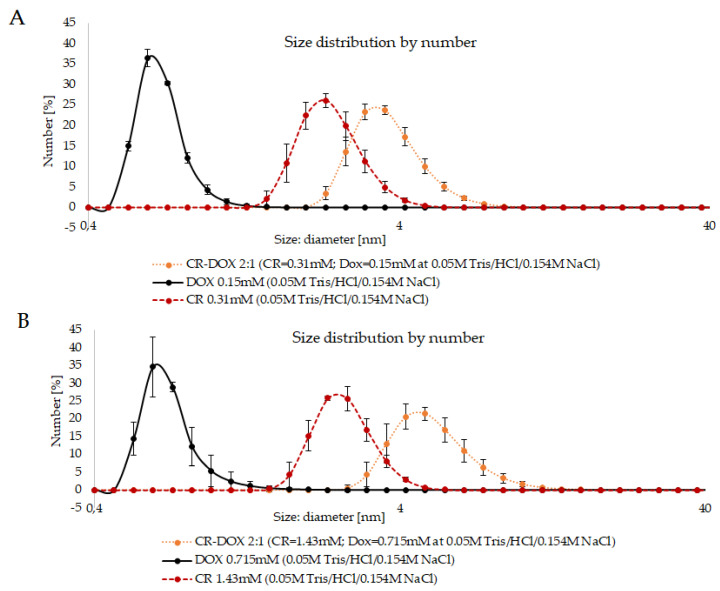
DLS analysis of CR-Dox complexes. The effect of concentration on the hydrodynamic diameter of CR, Dox, and CR-Dox complex. (**A**). lower concentration probes (final concentration of CR: 0.31 mM, Dox: 0.15 mM); (**B**). higher concentration probes (final concentration of CR: 1.43 mM, Dox: 0.715 mM).

DLS size analysis revealed well-defined peaks for CR-Dox complexes. The results indicate that the CR-Dox complex is bigger than free CR and free Dox. The size of the CR-Dox complex depends upon the initial concentrations of the components. The samples containing CR-Dox complexes at 2:1 CR:Dox molar ratio were compared with complexes formed at different component concentrations (lower: CR: 0.22 mg/mL, Dox: 0.09 mg/mL and higher: CR: 1 mg/mL, Dox: 0.83 mg/mL). In the case of free Dox and free CR, no effect of concentration on the hydrodynamic diameter was observed—for Dox it was 0.6 nm and for CR 2.3 nm for both concentrations. In the case of the CR-Dox complex, the size increased with increasing concentration, amounting to 3.6 nm for lower to 4.85 nm for the higher concentration (Figure 2). 

The above results show that Dox alone does not form supramolecular structures and that the dimensions of the supramolecular CR ribbon are not influenced by the concentration while in the case of CR-Dox complex, the size increases with concentration (for samples with the same proportion of the components). 

To explain the above results, it is necessary to clarify how the hydrodynamic diameter is read in the DLS analysis. In the case of spheroidal particles, it is just the diameter, but in the case of supramolecular CR, it is rather the width of the ribbon-like structure it creates in the solution. Both CR and Dox molecules contain charged groups, negative in the case of CR and positive in the case of Dox. As a result, supramolecular ribbons created by CR repel each other and the measured value of the hydrodynamic diameter is the same, even if the length of the ribbon differs, being higher at higher concentrations. A similar effect concerns Dox. In the case of CR-Dox complexes, the electrostatic interactions may stabilize the complex and the effect of electrostatic repulsion between separate ribbon-like structures disappears. This allows the individual ribbons to create more complex, tangled structures, registered in DLS analysis as the ones with higher hydrodynamic diameters. 

#### 3.1.2. DLS Analysis of CR-Dox Complexes—The Effect of Ionic Strength on Complex Size

**Figure 3:**
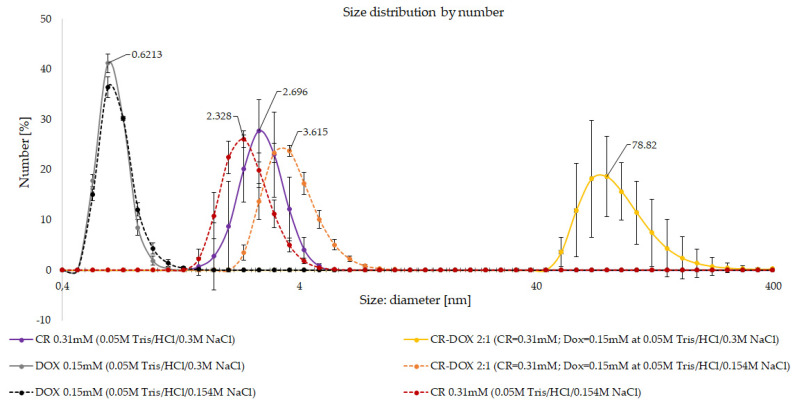
DLS analysis of CR-Dox complexes. The effect of ionic strength on the hydrodynamic diameter of CR, Dox, and CR-Dox complex. Lower concentration probes (final concentration of CR: 0.31 mM, Dox: 0.15 mM) at two different 0.05 M Tris/HCl buffers supplemented with: 0.154 M or 0.3 M NaCl.

The effect of ionic strength of the solution on hydrodynamic diameter of CR, Dox, and CR-Dox complex (2:1) was analyzed. Complexes were prepared in 0.05 M tris/HCl buffer with the addition of 0.154 M or 0.3 M NaCl. Higher ionic strength led to significantly increased size of CR-Dox complexes (3.6 vs. 78.82 nm) while it only slightly influenced the hydrodynamic diameter of CR ribbons (2.3 vs. 2.7 nm) and did not affect the results for Dox (0.6 nm for both NaCl concentrations, Figure 3).

To interpret the result, as in the case of the previous experiment, we need to note that in DLS analysis the hydrodynamic diameter value read by the DLS instrument reflects the diameter of the supramolecular ribbon formed by the analyzed molecules in the solution. As the ionic strength increases more charged groups become shielded due to the interaction with salt ions. For free CR the observed effect is rather small, but in the case of the CR-Dox complex, the neutralization of charges promotes the interaction between individual ribbon-like structures which produce bundles characterized by significantly higher hydrodynamic diameter. 

#### 3.1.3. Gel-Filtration Chromatography (BioGel P-10): Elution Volume (Ve) of CR, Dox, and CR-Dox

Gel filtration chromatography on BioGel P-10 columns was used to estimate the sizes of the complexes. Elution volumes (V_e_) for CR, Dox, and CR-Dox (2:1 molar ratio) complex were compared (Table 1). 

CR flows through the column very slowly and its elution volume is practically 100%, which may suggest adsorption to the BioGel bed. CR-Dox complex migrates faster than Dox alone indicating the formation of stable mixed SRLS created by CR and Dox. CR-Dox elution volume is 13% and Dox elution volume is 40%.

### 3.2. Interaction of Free CR and CR-Dox Co-Micelles with Proteins

#### 3.2.1. Agarose Gel Electrophoresis and Chromatographic Analysis

1. CR-Dox co-micelles form complexes with BSA (BSA-CR-Dox)

**Figure 4:**
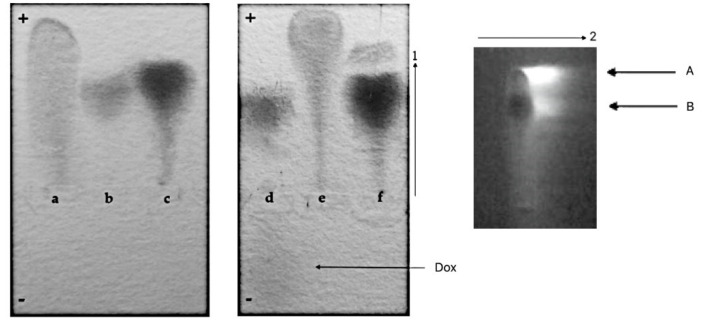
Complexes between BSA and CR-Dox (2:1)—replica on filter paper applied to bromophenol blue-stained gel (after agarose gel electrophoresis at pH 8.6); migration towards the anode (+): (**a**) CR, (**b**) BSA, (**c**) BSA-CR complex, (**d**) BSA and Dox (Dox can be seen as migrating towards the cathode (−), pointed by arrow), (**e**) CR-Dox complex, (**f**) BSA-CR-Dox (2:1) complex. The presence of Dox in the complex (BSA-CR-Dox) was confirmed chromatographically. The separation of CR-DOX mixtures was performed by Whatman 3 paper chromatography in butanol:acetic acid:water (5:1:4) solvent. Dox is seen as bright-orange fluorescence. For semiquantitative evaluation, DOX was eluted and the fluorescence was measured (emission signal at 550 nm upon excitation with a 470 nm laser beam) (the picture on the right; arrow no. 1 shows the direction of electrophoresis, and arrow 2 the direction of chromatography)—arrow (**A**) points to Dox released during chromatography from its complex with CR, while arrow (**B**) points to Dox released from the BSA-CR-Dox complex.

Albumin, a universal transporter protein, has the binding site for CR [10]. To check its ability to bind CR-Dox supramolecular ligand, the agarose gel electrophoresis employed as binding for CR-Dox (2:1 molar ratio) changes the net charge of the protein. As seen in Figure 4, the BSA-CR complex migrates faster than BSA (but slower than free CR). The fastest migration was observed for the CR-Dox complex. This can be explained by a strong interaction and high cohesion between molecules, leading to the increased dissociation of CR sulfonic groups and thus more acidic properties and higher electrophoretic mobility. Migration of BSA-CR-Dox complex is similar to that of BSA-CR, but two-dimensional separation (electrophoresis in the direction pointed by the arrow 1, followed by chromatography of the filter paper replica (arrow 2) reveals the presence of Dox in the complex. This confirms the presence of CR-mediated binding of Dox to BSA. Under the above experimental conditions, formation of stable complexes between BSA and Dox was not observed.

This experiment shows that this type of drug can be bound to albumin (BSA) via CR–a model SRLS.

2. CR-Dox co-micelles do not form complexes with Lλ

Conformational changes in the N-terminal loop of the immunoglobulin light chain λ (Lλ) heated up to 45 °C create the binding site for free CR [24]. Complexes with a defined stoichiometry (4 CR molecules per L chain monomer) can be visualized by agarose gel electrophoresis.

**Figure 5:**
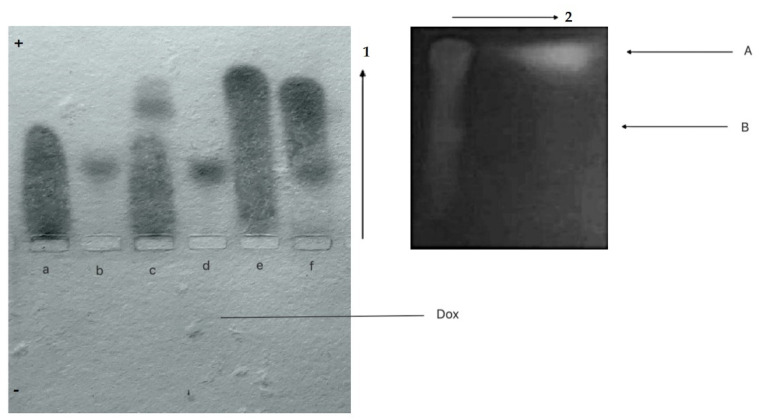
The possibility of creating complexes between Lλ and CR-Dox (2:1 molar ratio) was analyzed—replica on filter paper applied to bromophenol blue stained gel (after agarose gel electrophoresis at pH 8.6); migration towards the anode (+): (**a**) CR, (**b**) Lλ, (**c**) Lλ-CR complex, (**d**) Lλ-Dox mixture (Dox can be seen as migrating towards the cathode (−), pointed by arrow), (**e**) CR-Dox (molar ratio 2:1), (**f**) Lλ-CR-Dox (CR-Dox molar ratio 2:1); The absence of Dox in the Lλ-CR-Dox mixture was confirmed chromatographically. Arrow (**A**) points to Dox eluted from CR-Dox complex (fastest migration), arrow (**B**) points to the position of Lλ−CR complex (where Dox is absent).

The possibility of creating complexes between Lλ and CR-Dox co-micelles was analyzed. In agarose gel electrophoresis (pH 8.6), Dox migrates towards the cathode and free CR (a), Lλ chain (b), Lλ-CR complex (c), CR-Dox complexes (e) migrate towards the anode. CR forms a strong complex with Lλ, which migrates towards the anode twice as fast as free protein. For CR-Dox molar ratio 2:1, the formation of CR-Lλ complex is not observed, and all the CR is incorporated in CR-Dox faster-migrating complex, while Lλ migrates at the same speed as free unbound protein as in lane “b”. It suggests a strong competition between Lλ and doxorubicin for CR binding. All in all, preferentially the CR-Dox complex (migrating at the front) is formed first. This complex binds all the CR and thus Lλ-CR complex do not assembled, which is confirmed by the same migration distance of the protein as that of CR-free protein (f) (Figure 5). 

We conclude that Lλ binding site for SRLS created in the Lλ-chain at 45 °C can accommodate CR but not CR-Dox, suggesting that the binding site is specific and the incorporation of Dox changes the supramolecular properties of the CR.

#### 3.2.2. Gel-Filtration Chromatography (BioGel P-300): Elution Volumes of CR and CR-Dox Complexes with Proteins

Elution volumes (V_e_) of protein (BSA, Lλ) complexes with CR and CR-Dox were analyzed using gel filtration chromatography on BioGel P-300 columns. V_e_ values of free ligands and proteins (CR, Dox, BSA, Lλ), two-component complexes (BSA-CR, Lλ-CR), and three-component (triple) complex of BSA with CR-Dox co-micelle (BSA-CR-Dox) or a mixture of Lλ with CR-Dox co-micelle were compared (Table 2).

A low value of the elution volume for some complexes comparable to that of protein indicates the formation of large and stable co-micelles by e.g., CR and DOX. 

Free CR flows very slowly through the column, with tailing, which results from its adsorption to the BioGel. The elution volume of free BSA (V_e BSA free_) was 0.7 mL, while for BSA-CR it was V_e BSA-CR_ = 0.6 mL (both for separately determined CR and BSA). The BSA-CR complex shows a higher flow rate than ligand-free BSA. Simultaneous elution points to the formation of the BSA-CR complex. Elution volume for CR-Dox was V_e CR-Dox_ = 0.3 mL (both for separately determined CR and Dox). Simultaneous elution points to the formation of the CR-Dox co-micelle. So, we can conclude that CR-Dox at 2:1 molar ratio forms a stable, fast-migrating, and large complex which shows the migration rate twice as fast as that of BSA-CR and free BSA. The same elution volume for separately determined BSA, CR, and Dox was V_e BSA-CR-Dox_ = 0.3 mL. Simultaneous elution points to the binding of the CR-Dox co-micelle by BSA and formation of a stable, ternary complex with BSA.

The elution volume of free Lλ (V_e Lλ free_) was 1 mL. In the case of Lλ-CR, the V_e_ value differs for Lλ and CR (V_e__Lλ_ = 0.9; V_e__CR_ = 0.8). The separate elution from the column, but with increased flow rate for CR (as compared to the control, which is free CR), suggests that the complex is formed but dissociates during the flow through the column (which adsorbs CR). 

In the sample containing the mixture of Lλ and CR-Dox, different V_e_ values for CR-Dox co-micelle and Lλ were determined (V_e__Lλ_ = 0.8; V_e CR-Dox_ = 0.4). Separate elution points to the inability of Lλ to bind CR-Dox co-micelle.

Lλ migrates more slowly through the column than BSA due to its lower molecular weight. The differences in flow rates of Lλ-CR complex vs. free protein and free CR were observed. The mixture of Lλ and CR-Dox (2:1) did not produce a ternary complex (as in the case of BSA)–CR-Dox and Lλ were eluted separately from the column with V_e_ for CR-Dox equal to 0.4 and V_e_ for Lλ equal to 0.8. 

#### 3.2.3. CR Binds to Heat Aggregated Immunoglobulins G

**Figure 6:**
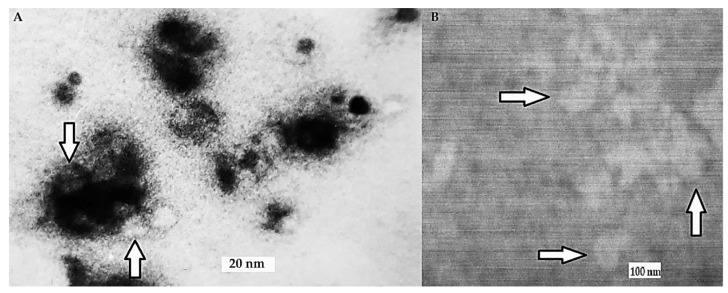
Complexes observed using TEM. (**A**). Heat aggregated immunoglobulins (HAI) with CR; (**B**). HAI without CR. Clusters of supramolecular Congo red can be distinguished around antibodies (darker spots). The images of immunoglobulins (brighter spots) are indicated by arrows.

HAI was dissolved with CR contrasted by AgNO_3_. The formed complexes were isolated using gel filtration chromatography on Sephadex G-200 thin layer. HAI surrounded by clusters of supramolecular Congo red were visible using transmission electron microscopy. HAI are more diffused in the presence of CR and form smaller clusters than HAI aggregated without CR. (Figure 6).

#### 3.2.4. Competition of BSA and HAI for Binding of the CR-RhoB Complexes (Congo Red-Rhodamine B) or CR-Dox (Congo Red-Doxorubicin) Complexes

Albumin has been shown to compete with HAI for binding of the CR-RhoB (or CR-Dox) and that it may eventually donate it to the immune complex model.

**Figure 7:**
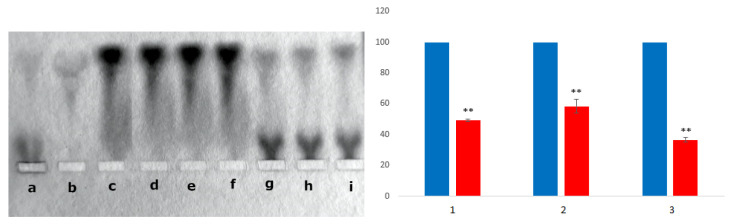
(**a**) HAI, (**b**) BSA, (**c**) BSA-CR, (**d**–**f**) BSA-CR-RhoB (1:1), (2:1), and (5:1) respectively, (**g**–**i**) BSA-CR-RhoB (1:1), (2:1), and (5:1) respectively after adding the same amount of HAI. The data in the graphs compare controls (blues bars) with results (red bars) and represent the mean ± SEM; test Student T. ** *p* < 0.01.

BSA-CR-RhoB complexes are formed when CR-RhoB is added to albumin. In electrophoresis, there is a clear difference in the location of BSA band (at the front, running faster) and HAI band (runs much slower, close to the loading site). After the addition of HAI to BSA-CR-RhoB complexes, CR-RhoB dissociates from BSA-CR-RhoB complexes and simultaneous binding of CR-RhoB to HAI is visible (Figure 7). Similar results were obtained with the drug-doxorubicin. These results indicate that albumin can be used as a carrier that binds other compounds, including CR-associated drugs. Such a drug can target the immune complex.

A loss of the CR-RhoB complex initially bound to albumin was observed after the addition of HAI in the amount of respectively: 50.6% in the case of the initially added CR:RhoB at molar ratio of 1:1 (compare “d” with “g” on the electrophoresis slab and with (1) on the histogram); 41.7% (for 2:1; compare “e” with „h” on the electrophoresis slab and (2) on the histogram) and 63.3% (for 5:1; compare “f” with “i” on the electrophoresis slab and (3) on the histogram). Complex binding to HAI is visible. The data in the graphs compare controls (blues bars) with results (red bars) and represent the mean ± SEM; test Student T. * *p* < 0.05, ** *p* < 0.01, *** *p* <0.001.

## 4. Discussion

The design of safe and reliable carriers for drugs is an important goal in the development of new therapies. The safe delivery of drugs using albumin, heat aggregated immunoglobulins, or antibodies as carriers in targeted immunotherapy, is already used as a solution which either supplements or substitutes the hitherto used therapies [36,37].

Earlier studies showed that stable mixed supramolecular assemblies can be formed by CR and other molecules characterized by planar, polyaromatic ring structure [30,31,32,33]. Supramolecular structure of Congo red dissociated by heating (80 °C) was found to form chaotic, frozen organization after rapid cooling, but it reorganizes upon gradual heating at about 25 °C deg forming standard ribbon-like structure and dissociates again above 60 °C [38]. In addition, the interaction of CR, as a model supramolecular ligand, with plasma albumin [10], partly unfolded immunoglobulin light chain [9], with HAI [6], and with antigen-complexed antibodies [25] was described. 

In this study, the analysis of the CR-drug complex (using DOX as an example) was extended, based on the previously conducted research [8,34,35,39,40]. Attempts were also made to introduce co-micelles formed from Congo red bound to Dox into all analyzed proteins. Moreover, the latest research using oil drop modeling has shown that not only free CR but also its complex with the drug can be bound to albumin. However, only free CR binds to the light chain while the CR-Dox complex is not bound [35]. 

The presented results provide new information about the properties of the SRLS-drug complexes and the capability of their interaction with proteins, and thus about their possible role in the delivery of drugs. It was shown that the L chain λ forms complexes with supramolecular CR but not with CR-Dox complexes (created at the 2:1 molar ratio). In the case of plasma, albumin complexes can be formed with both CR and CR-Dox. CR-Dox co-micelles were also bound by HAI, and additionally, the transfer of a part of the co-micelle-bound drug from the BSA-CR-Dox complex to HAI was observed.

The interaction of positively charged Dox with negatively charged CR molecules probably changes the regular, ribbon-like architecture of the supramolecular CR assembly. The strong interaction between CR and Dox results in the increased electrophoretic mobility of CR-Dox as compared to CR. The explanation for this phenomenon is the face-to-face alignment of Congo red molecules in supramolecular ribbon-like structures. In the electric field, these systems become uniquely oriented dipoles (due to the delocalization of pi electrons from stacked aromatic rings). Electron relocation affects the polar groups of Congo red, changing their dissociation constants and consequently their charge. Hence, accelerated electrophoretic migration towards the anode upon increasing CR concentration is observed, which is indicative of the concentration-dependent rise in acidity of Congo red. CR-Dox complexes are formed by the intercalation of doxorubicin between the Congo red molecules forming the ribbon. The mechanism of the dipole formation is the same and hence the acceleration of the CR-Dox complex migration towards the anode [34,41].

The CR-Dox complex is probably too large, and perhaps too compact, to enter the binding site in a partly unfolded L chain. This binding site is capable of accommodating the supramolecular CR micelle composed of just 4 CR molecules [6] and requires certain plasticity of SRLS which is a characteristic of CR alone and is limited (or absent) in CR-Dox complex. 

Therefore, the full analysis of protein complexes with a large CR-Dox complex could not be achieved using the partly unfolded L chain as a model. Supramolecular ligands, such as CR and CR-Dox can interact with proteins producing complexes in which some ligand molecules interact with the protein directly, while others are bound indirectly, as components of the SRLS. Such type of interaction would be possible in the case of HAI, where CR or CR-Dox could be localized also in-between immunoglobulins. It is thus possible that CR creates a kind of a scaffold structure capable of accommodating CR-Dox, and could work in the same way as in antigen-bound antibodies.

The potential of albumin to complex large-sized ligands (such as the CR-Dox complex) is much greater than that of the light chain model. Albumin, which has a ligand binding pocket, has greater ability to adapt this pocket to large ligands [35]. The structure of the ordered Congo red micelle changes significantly upon intercalation of other molecules (doxorubicin). The linear arrangement of the Congo red tape structure is likely to change, as indicated by the obtained results of the analysis using DLS and molecular filtration.

Albumin is one of the most often used drug carriers approved by the FDA, due to its high biocompatibility, availability, and accumulation in tissues that show high metabolic rate. It can also protect tissues against the harmful effects of the drugs it transports. Nab albumin nanoparticles (American Bioscience) have a diameter of 130–150 nm [42]. Albumin-based drug carriers are neither cytotoxic nor immunogenic, present optimum pharmacokinetics, are biodegradable, and easy to prepare [43,44,45]. Cancerous tissue presents increased capillary permeability and retention [EPR] and thus is more readily infiltrated by large particles, including albumin-based drug carriers. Here we show that native albumin can bind large ligands and can serve as a transporter for transport of SRLS-drug complexes.

Small molecules, like free Dox, can easily penetrate both healthy and diseased tissues while macromolecules (including albumin-drug conjugates or complexes) can easily infiltrate cancerous tissue but do not pass the endothelium of healthy ones. It increases the selective action of albumin-drug conjugates [46,47]. 

Albumin-CR-Dox complexes presented in this paper could serve as an alternative drug carrier. Due to the positive charge of Dox, its interaction with albumin is weak, and not observed in experimental systems presented here. Spectroscopy and docking results obtained by Agudelo et al. demonstrated that doxorubicin was able to bind to BSA and HSA via hydrophilic and hydrophobic interactions with more stable complexes created with human serum albumin than with bovine serum albumin. They also showed that drug-protein binding engaged several amino acid groups which were stabilized by a network of hydrogen bonds. The drug-protein interaction changed secondary structure of both bovine and human albumin causing partial destabilization of the protein. It can explain weak binding or no binding between BSA and Dox observed by us earlier [48]. 

Supramolecular systems, due to a wide range of their reactions with proteins and a variety of functional effects, become a new research tool in biology and pharmacology. The presented systems can be used as an alternative to the Nab technology (American Bioscience), consisting in mixing a hydrophobic drug suspended in an oil phase with an aqueous albumin solution and homogenization of the resulting mixture. The resulting albumin nanoparticles have a drug portion locked inside [42]. However, this technology does not work with hydrophilic, positively charged doxorubicin. The simplicity and speed of complex formation in the proposed system is also an interesting alternative to the previously tested combinations of albumin and doxorubicin [49]. On the other hand, drug delivery via CR that interacts with immune complexes is important due to the enhancing effect described in the literature [6]. 

Function-dependent Congo red binding to a protein like in the case when complexed with antigen-bound antibodies, consequently has a strong influence on this function. For instance, the affinity of antibodies forming immune complexes markedly increases in the presence of Congo red. Increasing the affinity of antibodies allows for the use of low-affinity antibodies in immune complexes, and an additional advantage is that an increased amount of drugs can be delivered in this way. The presented system offers a wide range of biomedical applications including drugs delivery to cancer cells.

## 5. Conclusions

To conclude, our results confirms that Congo red type supramolecular, self-assembled ribbon-like structures form complexes with the chemotherapeutic agent doxorubicin. CR-Dox are large-sized structures with properties different from the free CR. A model system composed of heated immunoglobulin light chain Lλ capable of CR binding, did not bind CR-Dox complexes. Heat aggregated immunoglobulins (HAI) and albumin were able to bind both free CR and CR-Dox complexes. Additionally albumin-bound CR-Dox complexes were transferred from albumin to HAI upon addition of HAI. This kind of interaction between CR-Dox and the described proteins, may in future become an important therapeutic system with the possibility of targeted drug transport and delivery. Supramolecular ribbon-like CR complexed with doxorubicin is a promising system in the treatment of cancers and may open new avenues for novel treatment strategies.

## Figures and Tables

**Table 1:** Elution volume for CR, Dox, and CR-Dox complex.

	CR	Dox	CR-Dox (2:1 Molar Ratio)
Elution volume (V_e_) (mL)	2.3	0.9	0.3

**Table 2:** Gel-filtration chromatography: elution volume (V_e_) of BSA, Lλ, CR, and Dox in mixtures of BSA-CR-Dox, Lλ-CR-Dox, CR-Dox, BSA-CR, Lλ-CR, and in free BSA and Lλ (Biogel P-300). The molar ratio of CR:Dox in all mixtures was 2:1. V_e DOX free_ = 2.1 mL (not shown).

	Mixtures						
Elution Volume (V_e_)	BSA-CR-Dox	Lλ-CR-Dox	CR-Dox	BSA-CR	Lλ-CR	BSA	Lλ
BSA (mL)	0.3	-	-	0.6	-	0.7	-
Lλ (mL)	-	0.8	-	-	0.9	-	1
CR (mL)	0.3	0.4	0.3	0.6	0.8	-	-
Dox (mL)	0.3	0.4	0.3	-	-	-	-
Complex formation:	**YES**	**NO**	**YES**	**YES**	±		

## Data Availability

The data presented in this study are available on request from the corresponding author.
